# The arginine methyltransferase PRMT5 and PRMT1 distinctly regulate the degradation of anti-apoptotic protein CFLAR_L_ in human lung cancer cells

**DOI:** 10.1186/s13046-019-1064-8

**Published:** 2019-02-08

**Authors:** Mingyue Li, Wentao An, Linyan Xu, Yidan Lin, Ling Su, Xiangguo Liu

**Affiliations:** 1 Shandong Provincial Key Laboratory of Animal Cell and Developmental Biology, Shandong University School of Life Sciences, Room N8-108, 72 Binhai Road, Qingdao, 266237 People’s Republic of China; 2 Shandong Provincial Collaborative Innovation Center of Cell Biology, School of Life Sciences, Shandong Normal University, Jinan, China; 3 Thoracic Surgery Department of West China Hospital, West China Medical School of Sichuan University, Province, Chengdu, 610064 Sichuan China

**Keywords:** CFLAR, PRMT1, PRMT5, ITCH, Apoptosis

## Abstract

**Background:**

CFLAR_L_, also known as c-FLIP_L_, is a critical anti-apoptotic protein that inhibits activation of caspase 8 in mammalian cells. Previous studies have shown that arginine 122 of CFLAR_L_ can be mono-methylated. However, the precise role of arginine methyltransferase of CFLAR_L_ remains unknown. PRMT5 and PRMT1, which are important members of the PRMT family, catalyze the transfer of methyl groups to the arginine of substrate proteins. PRMT5 can monomethylate or symmetrically dimethylate arginine residues, while PRMT1 can monomethylate or asymmetrically dimethylate arginine residues.

**Methods:**

Lung cancer cells were cultured following the standard protocol and the cell lysates were prepared to detect the given proteins by Western Blot analysis, and the protein interaction was assayed by co-immunoprecipitation (Co-IP) or GST pull-down assay. CFLAR_L_ ubiquitination level was evaluated by proteasomal inhibitor treatment combined with HA-Ub transfection and WB assay. PRMT1 and PRMT5 genes were knocked down by siRNA technique.

**Results:**

We show that PRMT5 up-regulated the protein levels of CFLAR_L_ by decreasing the ubiquitination and increasing its protein level. Additionally, PRMT1 down-regulated the protein level of CFLAR_L_ by increasing the ubiquitination and degradation. The overexpression of PRMT5 can inhibit the interaction between CFLAR_L_ and ITCH, which has been identified as an E3 ubiquitin ligase of CFLAR_L_, while overexpressed PRMT1 enhances the interaction between CFLAR_L_ and ITCH. Furthermore, we verified that dead mutations of PRMT5 or PRMT1 have the same effects on CFLAR_L_ as the wild-type ones have, suggesting it is the physical interaction between CFLAR and PRMT1/5 that regulates CFLAR_L_ degradation other than its enzymatic activity. Finally, we showed that PRMT5 and PRMT1 could suppress or facilitate apoptosis induced by doxorubicin or pemetrexed by affecting CFLAR_L_ in NSCLC cells.

**Conclusions:**

PRMT5 and PRMT1 mediate the distinct effects on CFLAR_L_ degradation by regulating the binding of E3 ligase ITCH in NSCLC cells. This study identifies a cell death mechanism that is fine-tuned by PRMT1/5 that modulate CFLAR_L_ degradation in human NSCLC cells.

**Electronic supplementary material:**

The online version of this article (10.1186/s13046-019-1064-8) contains supplementary material, which is available to authorized users.

## Introduction

CFLAR, which is a CASP8 and FADD-like apoptosis regulator, also known as c-FLIP, is an important regulatory protein in the extrinsic apoptotic pathway in mammalian cells. Several transcript variants encoding different isoforms have been reported. The short form, i.e., CFLAR_s_ (c-FLIP_S_), contains two N-terminal death effector domains (DED), whereas the long form, i.e., CFLAR_L_ (c-FLIP_L_), contains an additional pseudo-caspase domain in which the active center cysteine residue that confers the proteolytic activity of caspases is substituted by a tyrosine residue [1]. CFLAR_L_ can inhibit and prevent apoptosis by interfering with procaspase 8/10 for binding to the FADD domain to decrease caspase 8 activation. This binding prevents further death-inducing signaling complex (DISC) formation and subsequent activation of the caspase cascade [2]. High levels of CFLAR_L_ have been found in many different types of human cancers, and the excessive expression of the protein indicates a high degree of tumor malignancy [3]. In the clinic, CFLAR_L_ can be used as an independent adverse prognostic biomarker of colorectal cancer (CRC) [4]. Many chemotherapeutic agents have been shown to down-regulate CFLAR_L_ at the protein and mRNA level. Silencing its expression has been shown to facilitate apoptosis in chemotherapeutic agent-induced apoptosis. Therefore, CFLAR_L_ is a promising therapeutic target in some cancer treatments.

CFLAR_L_ can be regulated at both the transcriptional and post-translational level. NF-κB can induce the up-regulation of CFLAR_L_ at the mRNA and protein level and inhibit Fas, TNFR1 and TRAIL receptor-induced apoptosis [5]. c-MYC, FOXO3a, and c-Fos inhibit the transcription of CFLAR_L_ [6, 7]. Additionally, CFLAR_L_ has been shown to be down-regulated following treatment with compounds such as cycloheximide (CHX) and anisomycin [8, 9]. As the half-life of CFLAR_L_ is short, the ubiquitin-proteasome system plays an important role in regulating CFLAR_L_ degradation and stability. CFLAR_s_ is highly prone to ubiquitination and degradation likely due to its unique C-terminal tail [10]. The E3 ubiquitin ligase ITCH is thought to be responsible for CFLAR_L_ ubiquitination and degradation [11]. ITCH has also been shown to be an important regulator of CFLAR_s_ ubiquitination and stability [12, 13]. Furthermore, phosphorylation events play a vital role in the regulation of CFLAR_L_ protein levels; for example, the serine residue 273 of CFLAR_L_ is phosphorylated by AKT, which is important for the reduction of CFLAR_L_ via an ITCH-dependent mechanism [14]. The proteins that interact with CFLAR_L_ can also affect its stability. Recently, XRCC6 was shown to interact with, stabilize, and protect CFLAR_L_ from ubiquitin-proteasomal degradation [15]. XRCC6 usually forms a stable heterodimer consisting of two subunits (XRCC6 and XRCC5) [16]. Evidence suggests that XRCC proteins modulate ATM activity following DNA damage [17]. XRCC6 acts as an ATP-dependent single strand DNA helicase and has been found to play an important role in immune system disorders, aging and carcinogenesis [18].

Post-translational modifications, including phosphorylation, methylation, acetylation, ubiquitination, ADP-methylation, and SUMOylation, are highly important for the regulation of protein functioning in eukaryotic cells. Among these post-translational modifications, protein arginine methylation governs many cellular processes, such as cell growth, proliferation, differentiation and development [19]. The PRMT family members play a pivotal role in the regulation of the arginine methylation of both histones and other cellular proteins [20]. Three distinct types of methylated arginine residues, namely, omega-N^G^-mono-methylarginine (MMA), symmetric omega-N^G^,N^G^-dimethylarginine (sDMA), and asymmetric omega-N^G^,N^G^-dimethylarginine (aDMA), have been identified in mammalian cells [21]. The PRMT enzymes are classified into two groups depending on the type of modification they catalyze. Type I PRMT enzymes (PRMT1–4, PRMT6 and PRMT8) generate MMA and aDMA, whereas type II PRMT enzymes (PRMT5, PRMT7 and PRMT9) catalyze MMA and sDMA [22]. PRMT5 is a type II methyltransferase that modulates cell growth and transformation. PRMT5 hypermethylates histones H3R8 and H4R3 in promoters and restrains the cell cycle and tumor suppressor genes [23–25]. PRMT5 interacts with and methylates P53 at R333, R335, and R337 when DNA is damaged, inhibiting oligomerization between MDM2 and P53 [26, 27]. In particular, PRMT5 can co-localize with EGFR and regulate its monomethylation. R1175 methylation modulates EGF-induced EGFR trans- autophosphorylation at Y1173 [28].

PRMT1 was the first mammalian protein arginine methyltransferase identified [29]. Most protein arginine methylation is catalyzed by PRMT1 [30]. PRMT1 preferentially methylates arginine residues flanked by one or more glycine residues [31]. According to its three-dimensional structure, PRMT1 is active as a homodimer [32]. Moreover, PRMT1 has been reported to be a negative protein in Wnt/beta-catenin signaling by the methylation of Axin [33]. However, the mechanism by which the members of the protein arginine methyltransferase family, including PRMT5 and PRMT1, modulate apoptosis remains to be elucidated. Our work suggests that PRMT5 and PRMT1 regulate apoptosis by affecting CFLAR_L_ turnover.

## Methods

### Cell lines and cell culture

The lung cancer cell lines A549, H157, H460 and H1299 were cultured in RPMI 1640 supplemented with 5% (*v*/v) NBCS. The HEK293FT cell line was cultured in DMEM medium supplemented with 5% (v/v) newborn calf serum. All cell lines were originally obtained from the American Type Culture Collection (Manassas, VA) and maintained at 37 °C in a humidified atmosphere consisting of 5% CO_2_ and 95% air. The cells we used are routinely authenticated and tested for mycoplasma contamination.

### Antibodies and reagents

Doxorubicin and pemetrexed powder was purchased from Sigma Aldrich (Merck, Darmstadt, Germany) and diluted in dimethyl sulfoxide (DMSO). Stock solutions were stored at − 20 °C and diluted to the desired concentrations with growth medium before use. The PRMT5 (P4847), PRMT1 (G1544) and FLAG (F7425) antibodies were purchased from Sigma Aldrich (America). The CFLAR_L_ (ALX-804-961-0100) antibody was purchased from Enzo Biochem. The monomethyl arginine (8015S), CASP8 (9746 L) and PARP-1 (#9542) antibodies were purchased from Cell Signaling Technology (Boston, Massachusetts, US). The symmetric dimethyl arginine and asymmetric dimethyl arginine antibodies were purchased from Millipore.

### Western blot analysis

The preparation of whole-cell protein lysates and procedures used for the western blot analysis have been previously described [34]. The cells were harvested and rinsed with pre-chilled PBS. Then, the cells were lysed and centrifuged at 4 °C for 15 min. Samples of the whole-cell protein lysates (35 μg) were electrophoresed on a 12% denaturing polyacrylamide slab gel and then transferred to a polyvinylidene fluoride (PVDF) membrane by electroblotting. The proteins were probed with the appropriate primary antibodies and subsequently the secondary antibodies. Antibody binding was detected by an HRP system according to the manufacturer’s protocol.

### siRNA transfection

The siRNAs were synthesized by Boshang. The small interfering RNA (siRNA) duplexes used for the controls have been previously described [34]. The PRMT5 siRNA duplexes target the sequences 5’-GCCCAGUUUGAGAUGCCUU-3′ (#1) and 5’-CCGCUAUUGCACCUUGGAA-3′ (#2). The PRMT1 siRNA duplexes target the sequences 5’-CCACCAGCCCCGAGUCCCC-3′ (#1) and 5’-ACCGCAACUCCAUGUUUCA-3′ (#2). The negative control was 5’-UUCUCCGAACGUGUCACGU-3′. The siRNA transfections were carried out with the jet PRIME® siRNA Transfection Reagent (Polyplus) following the manufacturer’s instructions.

### Construction of the plasmids

The PRMT5 gene was amplified by PCR from H1299 cell genomic DNA using the following primers: MYC-PRMT5 sense: 5’-CGGATCCGCCGCCACCATGGAACAAAAACTCATCTCAGAAGAGGATCTGGCGGCGATGGCGGTCG-3′, PRMT5 sense: CGGATCCGCCGCCACCATGGCGGCGATGGCGGTCG antisense: 5’-CGAATTCCTAGAGGCCAATGGTATATGAG-3′; MYC-PRMT1 sense: 5’-CGGATCCGCCGCCACCATGGAACAAAAACTCATCTCAGAAGAGGATCTGGCGGCAGCCGAGGCCG-3′; PRMT1 (variant 1) sense: 5’-CGGATCCGCCGCCACCATGGCGGCAGCCGAGGCCG-3′ antisense: 5’-CGAATTCTCAGCGCATCCGGTAGTCGGT-3′; CFLAR_L_ sense: 5’-GGGTACCGCCGCCACCATGTCTGCTGAAGTCATCCAT-3’ CFLAR_L_ antisense: 5’-CCGCTCGAGTTATGTGTAGGAGAGGATAAG-3′; HA-ITCH sense: 5’-CGGATCCGCCGCCACCATGTACCCCTACGACGTGCCCGACTACGCCTCTGACAGTGGATCACAACT-3′; and ITCH sense: 5’-CGGATCCGCCGCCACCATGTCTGACAGTGGATCACAACT-3′ antisense: 5’-CGGGCCCTTACTCTTGTCCAAATCCTTCTG-3′. The plasmid construction procedures have been previously described [35, 36].

### Immunoprecipitation

Cells were lysed in lysis buffer (20 mM Tris-HCl, pH 7.5; 150 mM NaCl; 1 mM Na_2_EDTA; 1 mM EGTA; 2.5 mM sodium pyrophosphate; 1 mM β-glycerophosphate; 1 mM Na_3_VO_4_; 0.5% Triton) on ice for 30 min then purified via centrifugation for 15 min at 4 °C. The supernatants were incubated with antibody at 4 °C for 1 h. Then the mixture was incubated with protein A beads (ThermoFisher) at 4 °C for 2 h. The beads were washed twice with 1 ml of lysis buffer. 20 μl 2 × SDS buffer were added for elution (100 °C, 10 min). Samples were centrifuged for western blot analysis.

### GST pull-down assay

The HEK293FT cells were collected, lysed in immunoprecipitation lysis buffer and incubated on ice for 30 min. The lysates were purified via centrifugation for 15 min at 4 °C. The supernatants were incubated with glutathione sepharose at 4 °C overnight. The beads were washed 2 times with 900 μl of IP lysis (1% PIC) buffer, followed by incubation with 2XSDS at 100 °C for 10 min. Then, the beads were centrifuged for 5 min at room temperature. Finally, the supernatants were thoroughly collected for SDS-PAGE and western blot analysis.

### Flow cytometry analysis

Annexin V-FITC Apoptosis Detection Kit (Biobox Biotech, Nanjing, China) was used for cell apoptosis analysis according to the manufacture’s protocol.

### Statistical analysis

GraghPad Prism version 5.00 was used for statistical analysis. All data are presented as the mean ± SD. Differences between groups were identified using Student’s t-test. *P* < 0.05 was considered statistically significant.

## Results

### PRMT5 and PRMT1 regulate protein level of CFLAR_L_ and its sensitivity to chemotherapeutic agents

**Fig. 1:**
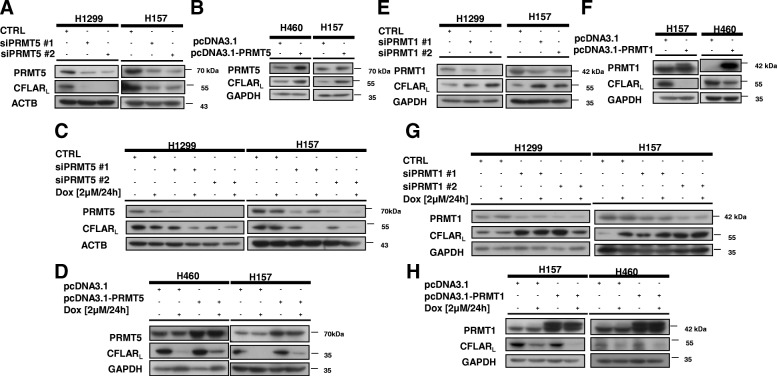
PRMT5 and PRMT1 regulate the CFLAR_L_ protein level and sensitivity to chemotherapeutic agents. **a** The indicated cells were seeded into a 6-well cell culture plate and transfected the following day with PRMT5 siRNAs for 48 h. **b** The indicated cells were seeded into a 6-well culture plate and transfected the following day with the pcDNA3.1 and pcDNA3.1-PRMT5 plasmids for 24 h. The cells were harvested for the preparation of the whole-cell protein lysates and subsequent western blot analysis. **c** The indicated cells were seeded into a 6-well cell culture plate and transfected with PRMT5 siRNAs on the second day. After 24 h, the cells were seeded again to ensure that the wells contained identical cell densities. Then, the cells were treated with 2 μmol/L doxorubicin for 24 h. The cells were harvested for the preparation of the whole-cell protein lysates and subsequent western blot analysis. **d** The indicated cells were seeded into a 6-well culture plate and transfected the following day with the pcDNA3.1 and pcDNA3.1-PRMT5 plasmids for 24 h. Then, the cells were treated with 2 μmol/L doxorubicin for 24 h. The cells were harvested for the preparation of the whole-cell protein lysates and subsequent western blot analysis. **d** Cells were seeded into a 6-well cell culture plate and transfected the following day with PRMT1 siRNAs for 48 h. **f** Cells were seeded into a 6-well culture plate and transfected the following day with the pcDNA3.1 and pcDNA3.1-PRMT1 plasmids for 24 h. The protein expression of CFLAR_L_ was calculated as described in (A). **g** The indicated cells were seeded into a 6-well cell culture plate and transfected with PRMT1 siRNAs on the second day. After 24 h, the cells were seeded again to ensure that each well contained identical cell densities. The protein expression of CFLAR_L_ was calculated as described in (c). Then, the cells were treated with 2 μmol/L doxorubicin for 24 h. **h** Cells were seeded into a 6-well culture plate and transfected the following day with the pcDNA3.1 and pcDNA3.1-PRMT5 plasmids for 24 h. Then, the cells were treated with 2 μmol/L doxorubicin for 24 h. The cells were harvested for the preparation of the whole-cell protein lysates and subsequent western blot analysis. ACTB or GAPDH was used as a loading control

Given that mass spectrometry analyses have revealed that the arginine 122 residue of CFLAR_L_ can be methylated [37], we sought to explore the role of the methylation of CFLAR_L_ in human lung cancer cells. Since arginine methyltransferases (PRMTs) can catalyze the monomethylation and symmetric or asymmetric dimethylation of histones and non-histones [38, 39], we examined whether PRMT5 and PRMT1 could alter the level of CFLAR_L_. We transfected NSCLC cells with siRNAs targeting the corresponding candidates and performed an immunoblot analysis to assess the expression status of CFLAR_L_. As shown in Fig. 1A, CFLAR_L_ expression was reduced by knocking down PRMT5 in human NSCLC cells. Consistently, the overexpression of PRMT5 led to an increase in CFLAR_L_ in the tested cells (Fig. 1B). Similar results were also obtained in NSCLC cells treated with doxorubicin; the protein level of CFLAR_L_ was lower in the PRMT5 siRNA-transfected cells than in the control cells (Fig. 1C). The overexpression experimental results are consistent with the results mentioned above (Fig. 1D). Thus, doxorubicin alters CFLAR_L_ levels in cells when PMRT5 is silenced in NSCLC cells. Similarly, we blocked PRMT1 expression using PRMT1 siRNA in H157 and H1299 cells, and the western blot analysis showed that the expression of CFLAR_L_ was much increased (Fig. 1E). Consistently, the overexpression of PRMT1 led to a reduction in the CFLAR_L_ levels (Fig. 1F). In NSCLC cells treated with doxorubicin, the protein level of CFLAR_L_ was considerably higher in the PRMT1 siRNA-transfected cells than in the control siRNA-transfected cells (Fig. 1G). This effect was also observed in the PRMT1-overexpressed cells (Fig. 1H). Thus, the inhibition of PRMT1 could protect CFLAR_L_ from doxorubicin-mediated degradation.

### Both PRMT5 and PRMT1 can interact with CFLAR_L_

**Fig. 2:**
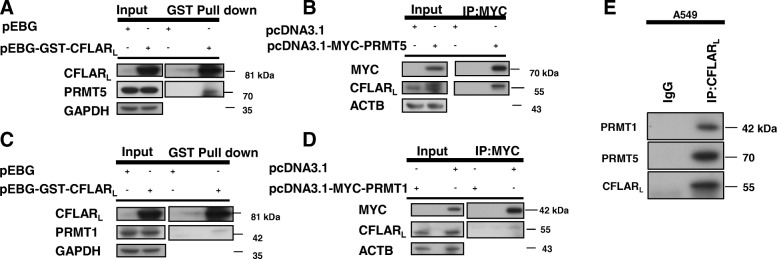
Both PRMT5 and PRMT1 interact with CFLAR_L_. **a** 293FT cells were transfected with the pEBG and pEBG-CFLAR_L_ plasmids. The cells were harvested and prepared for the GST pull-down assay after 24 h, and the CFLAR_L_ protein was detected by western blotting. **b** Cells were transfected with the pcDNA3.1 and pcDNA3.1-MYC-PRMT5 plasmids. The cells were harvested and prepared for the immunoprecipitation assay after 24 h. The lysate supernatant was incubated with the MYC antibody for 1 h, followed by direct incubation with protein-A (1:1 mix) beads at 4 °C overnight. The precipitated proteins were analyzed by a western blot assay. **c** The same as described in (A). **d** Cells were transfected with the pcDNA3.1 and pcDNA3.1-MYC-PRMT1 plasmids. The cells were harvested and prepared for the immunoprecipitation assay after 24 h. The lysate supernatant was incubated with the MYC antibody for 1 h and then directly with protein-A beads at 4 °C overnight. The precipitated proteins were analyzed by western blot analysis. GAPDH was used as a loading control. **e** IP assay using CFLAR_L_ antibody was conducted in A549 cells and endogenous PRMT1/5 was detected

To examine whether PRMT5 and PRMT1 interact with CFLAR_L_, we carried out GST pull-down assays by co-transfecting PRMT5 or PRMT1 plasmids and pEBG-GST-CFLAR_L_ in 293FT cells. The results show that CFLAR_L_ interacts with both PRMT5 and PRMT1. To further verify the physical interaction between PRMT5 and CFLAR_L_, 293FT cells were transfected with plasmids carrying control plasmid pcDNA3.1 or MYC-tagged PRMT5. After immunoprecipitation with the MYC antibody, MYC-PRMT5 was pulled down. The western blot analysis showed that PRMT5 certainly interacts with CFLAR_L_ (Fig. 2A, B). We used similar methods to verify the relationship between PRMT1 and CFLAR_L_ (Fig. 2C, D). The endogenous interaction between CFLAR_L_ and PRMT1/5 was also examined (Fig. 2E). To determine the region in PRMT5 and PRMT1 critical for binding CFLAR_L_, we constructed several deletion mutants of PRMT5 and PRMT1. The full-length CFLAR_L_ mainly bound to the TIM barrel domain and oligomerization domain of PRMT5 (c Fig. S1A). And, the full-length CFLAR_L_ could bind two truncated parts of PRMT1 (Additional file 1: Figure S1B). Further co-immunoprecipitation analyses revealed that the full-length PRMT5 and PRMT1 could bind the caspase-like domain but not the DED domain of CFLAR_L_ (Additional file 1: Figure S1C, S1D). Thus, the P20 and P12 domains (caspase-like domain) of CFLAR_L_ are important for the interaction between PRMT5/PRMT1 and CFLAR_L_.

### PRMT1 and PRMT5 regulates CFLAR_L_ degradation independently of their enzymatic activity

**Fig. 3:**
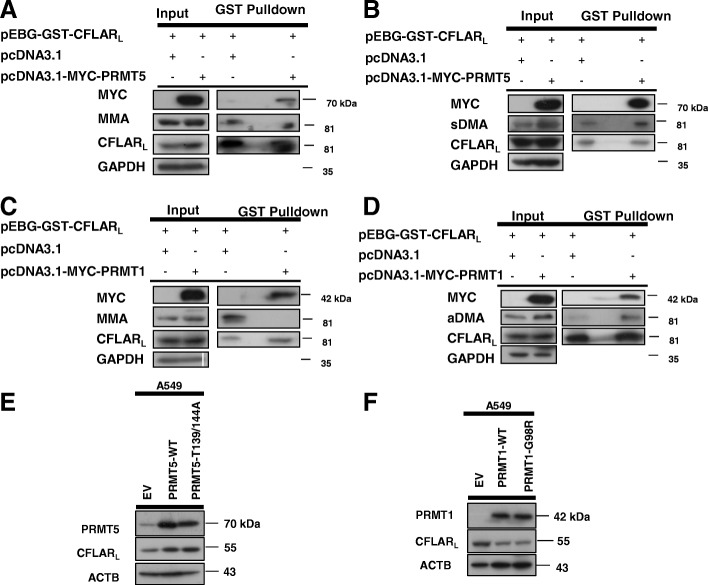
PRMT1 and PRMT5 regulates CFLARL independently of their enzymatic activity. **a** H1299 cells were transfected with the pEBG-CFLAR_L_ plasmids and co-transfected with pcDNA3.1 or pcDNA3.1-MYC-PRMT5. The cells were harvested and prepared for the GST pull-down assay after 24 h, and the arginine monomethylation of the CFLAR_L_ protein was detected by western blotting. **b** The symmetric arginine dimethylation of the CFLAR_L_ protein was detected by western blotting. **c** Cells were transfected with the pEBG-CFLAR_L_ plasmids and co-transfected with pcDNA3.1 or pcDNA3.1-MYC-PRMT1. The cells were harvested and prepared for the GST pull-down assay after 24 h, and the arginine monomethylation of the CFLAR_L_ protein was detected by western blotting. **d** The asymmetric arginine dimethylation of the CFLAR_L_ protein was detected by western blotting. **e** and **f** A549 cells were seeded in 6-well plates. PRMT5-WT and PRMT5-T139/144A plasmids or PRMT1-WT and PRMT1-G98R plasmids were transfected for 24 h, while pcDNA3.1 was transfected as control. Cells were harvested for western blot analysis

Considering that PRMT5 and PRMT1 belong to a major class of enzymes that catalyze the three types of arginine methylation reactions, we examined the types of CFLAR_L_ arginine methylation in vivo. Thus, we carried out pull-down assays by overexpressing pEBG-GST-CFLAR_L_ in H1299 cells. As shown in Fig. 3A, the level of monomethyl arginine in CFLAR_L_ was decreased after the PRMT5 overexpression. However, the level of symmetric dimethyl arginine in CFLAR_L_ was increased after PRMT5 was overexpressed (Fig. 3B). Then, we tested the methylation of CFLAR_L_ after PRMT1 was overexpressed. By performing the pull-down assay, we found that the level of monomethyl arginine in CFLAR_L_ was also decreased (Fig. 3C); however, the level of asymmetric dimethyl arginine of CFLAR_L_ was increased (Fig. 3D).

In order to characterize whether the arginine methylation or the physical interaction of CFLAR_L_ and PRMT1/5 regulates the degradation of CFLAR_L_, we constructed the plasmids containing PRMT1 or PRMT5 genes with dead mutations as described before [40, 41]. The plasmids are designated as pcDNA3.1-PRMT5-T139/144A and pcDNA3.1-PRMT1- G98R, respectively. As shown in Fig. 3E, CFLAR_L_ was upregulated after overexpression of both wild-type PRMT5 and its dead mutation in A549 cells. Similarly, CFLAR_L_ was downregulated after overexpression of both wild-type PRMT1 and its dead mutation in A549 cells (Fig. 3F), suggesting it is the physical interaction of CFLAR_L_ and PRMT1/5 that regulates CFLARL degradation other than its enzymatic activity.

### PRMT1 and PRMT5 regulate poly-ubiquitination of CFLAR_L_ by affecting the interaction between CFLAR_L_ and ITCH

**Fig. 4:**
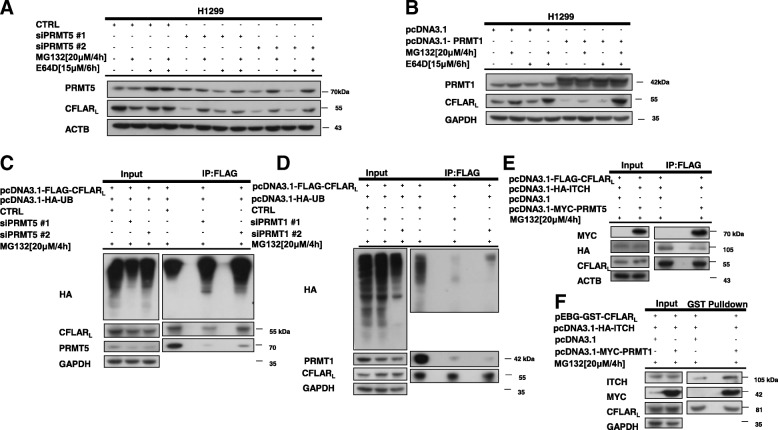
PRMT5 and PRMT1 regulate the polyubiquitination of CFLAR_L_ by affecting the interaction between CFLAR_L_ and ITCH. **a** H1299 cells were seeded into a 6-cm cell culture plate and transfected with PRMT5 siRNAs on the second day. After 24 h, the cells were seeded again into a 6-well cell culture plate to ensure that each well contained identical cell densities. Then, the cells were treated with 20 μmol/L MG132 for 4 h or 15 μmol/L E64D for 6 h. The cells were harvested for the preparation of the whole-cell protein lysates and subsequent western blot analysis. **b** H1299 cells were seeded into a 6-cm cell culture plate and transfected with the PRMT1 plasmid on the second day. After 24 h, the cells were seeded again into a 6-well cell culture plate to ensure that each well contained identical cell densities. Then, the cells were treated with 20 μmol/L MG132 for 4 h or 15 μmol/L E64D for 6 h. The cells were harvested for the preparation of the whole-cell protein lysates and subsequent western blot analysis. **c** Cells were transfected with the pcDNA3.1-FLAG-CFLAR_L_ or pcDNA3.1-HA-UB plasmids and co-transfected with ctrl siRNA or PRMT5 siRNA. The cells were harvested and prepared for the IP assay after 16 h. Then, the cells were treated with 20 μmol/L MG132 for 4 h. The polyubiquitination level of the CFLAR_L_ protein was detected by western blotting. **d** Cells were transfected with the pcDNA3.1-FLAG-CFLAR_L_ or pcDNA3.1-HA-UB plasmids and co-transfected with ctrl siRNA or PRMT1 siRNA. The cells were harvested and prepared for the IP assay after 16 h, and then, the cells were treated with 20 μmol/L MG132 for 4 h. The polyubiquitination level of the CFLAR_L_ protein was detected by western blotting. **e** Cells were transfected with the pcDNA3.1-FLAG-CFLAR_L_ /pcDNA3.1-HA-ITCH plasmids and co-transfected with pcDNA3.1 or pcDNA3.1-MYC-PRMT5. The cells were harvested and prepared for the co-IP assay after 24 h. The protein levels of CFLAR_L_ and HA were detected by western blotting. **F** HEK293FT cells were transfected with the pEBG-GST-CFLAR_L_/ pcDNA3.1-HA-ITCH plasmids and co-transfected with pcDNA3.1 or pcDNA3.1-MYC-PRMT1. The cells were harvested and prepared for the GST pull-down assay after 24 h. The protein levels of CFLAR_L_ and ITCH were detected by western blotting. ACTB or GAPDH expression was detected as a loading control

Recent studies have reported that CFLAR_L_ is degraded predominately by the ubiquitin-proteasome degradation pathway as well as lysosomal pathway [42, 43]. Since PRMT5 and PRMT1 could modulate the protein levels of CFLAR_L_, we examined whether they affect CFLAR_L_ ubiquitination and degradation. Therefore, we blocked PRMT5 expression using PRMT5 siRNA in H1299 cells and then treated the cells with 20 μmol/L MG132 for 4 h or 15 μmol/L E64D for 6 h. MG132 is a specific, potent, reversible and cell-permeable proteasome inhibitor that reduces the degradation of ubiquitin-conjugated proteins in mammalian cells via the 26S complex. E64D, which is a synthetic analog of E-64 and an ethyl ester of E-64C, is an irreversible, membrane-permeable inhibitor of lysosomal and cytosolic cysteine proteases. E-64D inhibits calpain and the cysteine protease cathepsins F, K, B, H, and L [44, 45]. Our data indicated that the silencing of PRMT5 by small interfering RNA could down-regulate the protein levels of CFLAR_L_. The PRMT5 knockdown-mediated CFLAR_L_ degradation was prevented by the addition of the proteasome inhibitor MG132 (Fig. 4A). Similarly, we found that PRMT1 also decreased the protein expression of CFLAR_L_ after PRMT1 was overexpressed (Fig. 4B). Subsequently, we examined the ubiquitination level of CFLAR_L_, and our results suggest that the PRMT5 knockdown promoted the polyubiquitination level of CFLAR_L_ (Fig. 4C). In addition, the polyubiquitination level of CFLAR_L_ decreased after PRMT1 was knocked down (Fig. 4D). ITCH has been reported to be the E3 ubiquitin ligase of CFLAR_L_. To determine whether PRMT5 and PRMT1 regulate CFLAR_L_ by affecting the interaction between CFLAR_L_ and ITCH, we carried out GST pull-down assays and co-immunoprecipitation assays. Our data indicate that the overexpression of PRMT5 reduced the interaction between CFLAR_L_ and ITCH (Fig. 4E). After we overexpressed PRMT1, the interaction between CFLAR_L_ and ITCH was enhanced (Fig. 4F). Taken together, we conclude that PRMT5 and PRMT1 regulate the ubiquitination of CFLAR_L_ by affecting the interaction between CFLAR_L_ and ITCH.

### PRMT5 and PRMT1 regulate degradation of CFLAR_L_

**Fig. 5:**
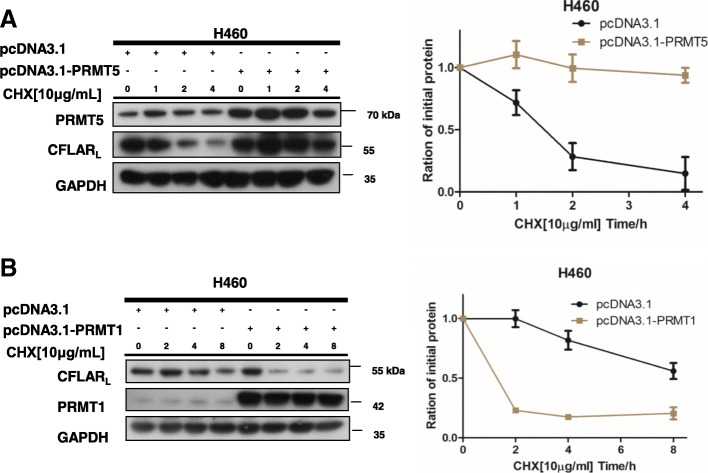
PRMT5 and PRMT1 regulate the turnover of CFLAR_L_. **a** H460 cells were transfected with the pcDNA3.1 and pcDNA3.1-PRMT5 plasmids, and after 24 h, the cells were seeded again into a 6-well cell culture plate to ensure that each well contained identical cell densities. The cells were treated with 10 μg/ml CHX for 0 h, 1 h, 2 h, and 4 h. Then, the cells were harvested, and whole-cell protein lysates were prepared to detect the expression of CFLAR_L_. **b** H460 cells were transfected with the pcDNA3.1 and pcDNA3.1-PRMT1 plasmids. Regarding the subsequent experiments, refer to (A). ACTB or GAPDH expression was detected as a loading control. The dynamics of CFLAR_L_ turnover were calculated using software Image J, and the curves were drawn with excel

Considering that the polyubiquitination level of CFLAR_L_ is regulated by PRMT5 and PRMT1, we explored whether they regulated CFLAR_L_ degradation. We conducted cycloheximide chase assays in H460 cells. We used 10 μg/ml cycloheximide (CHX) to block protein synthesis and examined CFLAR_L_ protein turnover in control and PRMT5 or PRMT1 plasmid-transfected cells for the indicted time points. As shown in Fig. 5A and B, the half-life of CFLAR_L_ was significantly lenthened in the PRMT5 overexpressed cells compared with that in the control cells, while the half-life of CFLAR_L_ was shortened in the PRMT1 overexpressed cells compared with that in the control cells. Taken together, we conclude that PRMT5 and PRMT1 contribute to the turnover of CFLAR_L_ as regulators.

### PRMT5 and PRMT1 modulate caspase 8 cleavage and apoptosis induced by the anti-cancer drugs in NSCLC cells

**Fig. 6:**
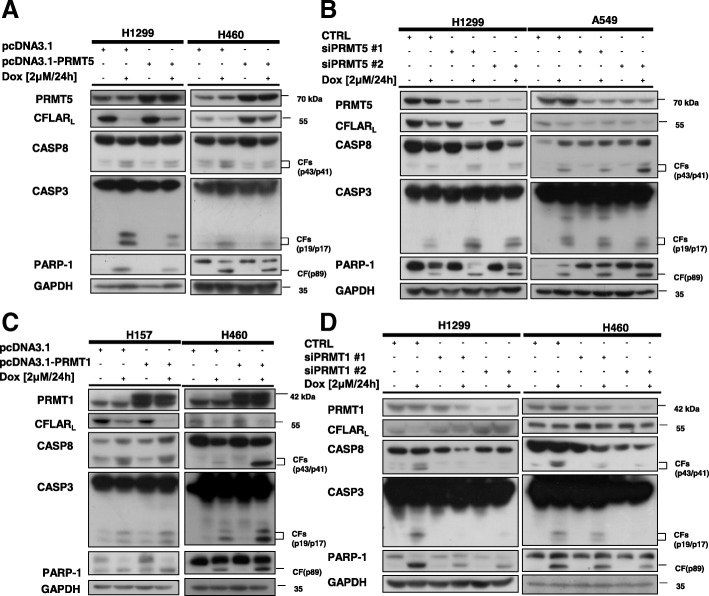
PRMT5 and PRMT1 affect NSCLC cell apoptosis induced by doxorubicin. **a** H460 and H1299 cells were seeded into 6-well cell culture plates and transfected with the PRMT5 plasmid on the second day. After 24 h, the cells were seeded again to ensure that each well contained identical cell densities. The cells were then treated with 2 μmol/L doxorubicin for 24 h. The cells were harvested for the preparation of the whole-cell protein lysates and subsequent western blot analysis. GAPDH expression was detected as a loading control. **b** A549 and H1299 cells were seeded into 6-well cell culture plates and transfected with PRMT5 siRNA on the second day. After 24 h, the cells were seeded again to ensure that each well contained identical cell densities. Then, the cells were treated with 2 μmol/L doxorubicin for 24 h. The cells were harvested for the preparation of the whole-cell protein lysates and subsequent western blot analysis. **c** H157 and H460 cells were seeded into 6-well cell culture plates and transfected with the PRMT1 plasmid on the second day. After 24 h, the cells were seeded again to ensure that each well contained identical cell densities. Then, the cells were treated with 2 μmol/L doxorubicin for 24 h. The cells were harvested for the preparation of the whole-cell protein lysates and subsequent western blot analysis. **d** H460 and H1299 cells were seeded into 6-well cell culture plates and transfected with PRMT1 siRNA on the second day. After 24 h, the cells were seeded again to ensure that each well contained identical cell densities. Then, the cells were treated with 2 μmol/L doxorubicin for 24 h. The cells were harvested for the preparation of the whole-cell protein lysates and subsequent western blot analysis. GAPDH expression was detected as a loading control

CFLAR_L_ is known as a crucial anti-apoptotic regulator that suppresses death receptor-induced apoptosis by interfering with the processing of procaspase 8 in DISC [20]. Therefore, we explored the effect of PRMT1/5 on caspase cleavage and activation. We overexpressed PRMT5 in lung cancer cells for 24 h and then treated the cells with 2 μmol/L doxorubicin for 24 h. Several apoptosis-related proteins were examined by performing a western blot analysis. The data showed that CFLAR_L_ was up-regulated, and weak cleavage of CASP8, CASP3 and PARP was induced by doxorubicin in the PRMT5-overexpressed cells after the exposure to doxorubicin (Fig. 6A), indicating that PRMT5 protects cells from caspase activation and apoptosis induced by doxorubicin. Coincidently, we also knocked down PRMT5 expression using siRNA in lung cancer cells. The data demonstrated that CFLAR_L_ was down-regulated, and caspase activation induced by doxorubicin was strengthened after PRMT5 was silenced (Fig. 6B). We also overexpressed PRMT1 in lung cancer cells for 24 h and then treated the cells with 2 μmol/L doxorubicin for 24 h. The western blot analysis showed a high cleavage of CASP8, CASP3 and PARP induced by doxorubicin in the PRMT1-overexpressed cells after exposure to doxorubicin, indicating that PRMT1 promotes caspase activation after doxorubicin exposure (Fig. 6C). In addition, we knocked down PRMT1 and found that CFLAR_L_ was up-regulated and that cellular apoptosis induced by doxorubicin was markedly weakened after PRMT1 was silenced (Fig. 6D). We also examined the percentage of pemetrexed-induced apoptotic cells after PRMT5 and PRMT1 alteration using FACS technique. Cellular apoptosis was reduced after PRMT5 overexpression or PRMT1 knockdown, and cellular apoptosis was increased after PRMT5 knockdown (Fig.S2). Taken together, we conclude that PRMT5 and PRMT1 truly affect apoptosis induced by anti-cancer drugs doxorubicin and pemetrexed in NSCLC cells.

### PRMT5 and PRMT1 modulate apoptosis in NSCLC cells by regulating protein levels of CFLAR_L_

**Fig. 7:**
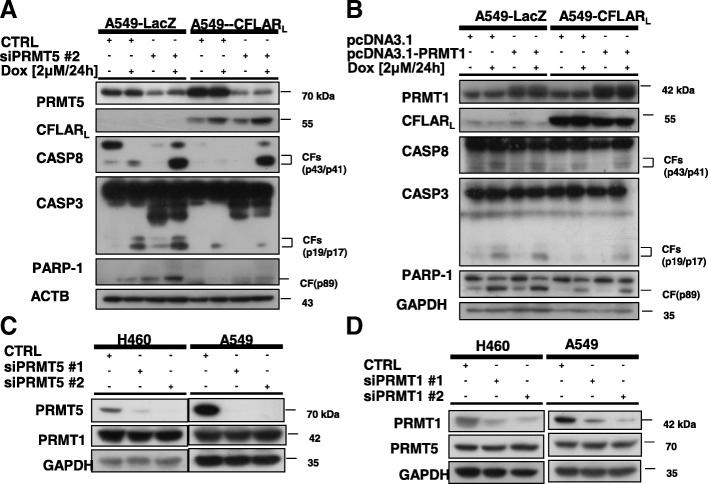
PRMT5 and PRMT1 affect NSCLC cellular apoptosis by regulating the protein levels of CFLAR_L_. **a** A549-LacZ cells and A549-CFLAR_L_ cells were seeded into 6-well cell culture plates and transfected with PRMT5 siRNA on the second day. After 24 h, the cells were seeded again to ensure that each well contained identical cell densities. Then, the cells were treated with 2 μmol/L doxorubicin for 24 h. The cells were harvested for the preparation of the whole-cell protein lysates and subsequent western blot analysis. **b** A549-LacZ cells and A549-CFLAR_L_ cells were seeded into 6-well cell culture plates and transfected with the pcDNA3.1-PRMT1 plasmid on the second day. After 24 h, the cells were seeded again to ensure that each well contained identical cell densities. Then, the cells were treated with 2 μmol/L doxorubicin for 24 h. The cells were harvested for the preparation of the whole-cell protein lysates and subsequent western blot analysis. **c** H460 cells and A549 cells were transfected with control siRNA or PRMT5 siRNA. After 48 h, the cells were harvested and lysed for the western blot analysis. **D** H460 cells and A549 cells were transfected with control siRNA or PRMT1 siRNA. After 48 h, the cells were harvested and lysed for the western blot analysis

Since PRMT5 and PRMT1 could modulate apoptosis and CFLAR_L_ levels, we considered whether PRMT5 and PRMT1 affect human NSCLC cell apoptosis by regulating CFLAR_L_ expression. Accordingly, we constructed an A549 cell line that could stably overexpress CFLAR_L_. The cleavage of pro-caspases and PARP-1 in the PRMT5 knockdown A549-CFLAR_L_ cells was weaker than that in the control knockdown cells after treatment with doxorubicin, suggesting that CFLAR_L_ could prevent cancer cells from PRMT5-knockdown-enhanced apoptosis after doxorubicin treatment (Fig. 7A). Moreover, we overexpressed PRMT1 in A549-LacZ cells and A549-CFLAR_L_ cells and simultaneously treated the cells with doxorubicin. We found that the enhanced apoptosis caused by the PRMT1 overexpression was suppressed in the A549-CFLAR_L_ cells compared with that in the A549-LacZ cells (Fig. 7B). Taken together, we conclude that PRMT5 and PRMT1 affect apoptosis by regulating the CFLAR_L_ level in NSCLC cells. In addition, we found that the PRMT5 knockdown did not affect the expression of PRMT1, and vice versa (Fig. 7C, D).

### PRMT5 competes with PRMT1 for binding to CFLAR_L_

**Fig. 8:**
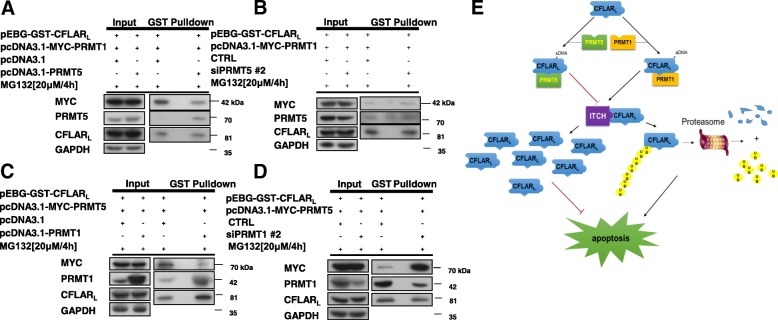
PRMT5 competes with PRMT1 for binding to CFLAR_L_. **a** H1299 cells were transfected with the pEBG-GST-CFLAR_L_ or pcDNA3.1-MYC-PRMT1 plasmids and co-transfected with pcDNA3.1 or pcDNA3.1–PRMT5. Then, the cells were treated with 20 μmol/L MG132 for 4 h. The cells were harvested and prepared for the GST pull-down assay. The protein levels of CFLAR_L_ and MYC were detected by western blotting. **b** Cells were transfected with the pEBG-GST-CFLAR_L_ or pcDNA3.1-MYC-PRMT1 plasmids and co-transfected with control siRNA or PRMT5 siRNA. Regarding the subsequent experiments, refer to (A). **c** Cells were transfected with the pEBG-GST-CFLAR_L_ or pcDNA3.1-MYC-PRMT5 plasmids and co-transfected with pcDNA3.1 or pcDNA3.1–PRMT1. Then, the cells were treated with 20 μmol/L MG132 for 4 h. The cells were harvested and prepared for the GST pull-down assay. The protein levels of CFLAR_L_ and MYC were detected by western blotting. **d** Cells were transfected with the pEBG-GST-CFLAR_L_ or pcDNA3.1-MYC-PRMT5 plasmids and co-transfected with control siRNA or PRMT1 siRNA. Regarding the subsequent experiments, refer to (C). **e** Summary of PRMT5-regulated and PRMT1-regulated apoptosis signaling pathways in NSCLC cells

After verifying that both PRMT5 and PRMT1 interact with CFLAR_L_, we contemplated whether PRMT5 competes with PRMT1 for binding to CFLAR_L_. Thus, we conducted GST pull-down assays. Our data indicate that the overexpression of PRMT5 could attenuate the interaction between CFLAR_L_ and PRMT1 (Fig. 8A). Silencing the expression of PRMT5 by RNAi facilitated the interaction between CFLAR_L_ and PRMT1 (Fig. 8B). Similarly, after PRMT1 was overexpressed, the interaction between CFLAR_L_ and PRMT5 was attenuated (Fig. 8C). The PRMT1 knockdown increased the interaction between CFLAR_L_ and PRMT5 (Fig. 8D).

## Discussion

In the present study, we report a new finding that PRMT5 and PRMT1, which are both arginine methyltransferases, distinctly regulate the turnover of CFLAR_L_ in human NSCLS cells independently of its enzymatic activity. CFLAR_L_ is a critical regulator of apoptosis induction and drug resistance in multiple cancers, such as colon, rectum and lung cancer, and has been considered a potential anti-cancer molecular target. Thus, investigating its molecular mechanisms is particularly meaningful. Our present work verified that PRMT5 and PRMT1 could affect its ubiquitination, degradation. Although overexpression of PRMT5 catalyzed CFLAR_L_ to produce arginine symmetric dimethylation, and PRMT1 catalyzed CFLAR_L_ to produce arginine asymmetric demethylation, the dead mutations of PRMT1 and PRMT2 had the same effect on the CFLAR_L_ level as the wild-type ones had, suggesting it is the physical interaction between CFLAR and PRMT1/5 that regulates CFLARL degradation other than its enzymatic activity.

Several reports have shown that PRMT5 overexpression is associated with hyper-proliferation and apoptosis resistance in cancer cells, and the inhibition of its activity leads to the repression of cancer genes and slows growth [46, 47]. PRMT5 plays essential roles in the regulation of protein function, quality control and signal transduction [48]. Our results show that PRMT5 enhance CFLAR_L_ stability. The link between PRMT5 and CFLAR_L_ protein longevity is novel and reasonable.

In our study, we found that PRMT5 and PRMT1 regulate the protein level of CFLAR_L_ and the sensitivity to chemotherapy drugs. The experiments showed that PRMT5 knockdown or PRMT1 overexpression promoted cell apoptosis induced by doxorubicin and pemetrexed. The PRMT5 overexpression and PRMT1 knockdown inhibited apoptosis induced by chemotherapeutic agents. Furthermore, cellular apoptosis induced by doxorubicin along with the PRMT5 knockdown or PRMT1 overexpression could be inhibited by the exogenous replenishment of CFLAR_L_, suggesting that PRMT5 and PRMT1 affect apoptosis by regulating the protein level of CFLAR_L_ in NSCLC cells. We speculated how these two arginine methyltransferases contributed to apoptosis induced by chemotherapeutic agents. Therefore, we verified that both PRMT5 and PRMT1 bound CFLAR_L_, regulating CFLAR_L_ by affecting its ubiquitin-proteasome degradation. The level of CFLAR_L_ polyubiquitination is increased after overexpressing PRMT1 or knocking down PRMT5 in HEK293FT cells. Our data showed that PRMT5 and PRMT1 were involved in the interaction between CFLAR_L_ and the E3 ligase ITCH. The PRMT5 silencing and PRMT1 overexpression enhanced the interaction between CFLAR_L_ and ITCH, leading to an altered ubiquitination level and, eventually, the degradation of CFLAR_L_.

## Conclusions

In summary, in the present study, we explored the role of the interaction between CFLAR_L_ and PRMT5/PRMT1 in apoptosis in NSCLC cells. We also demonstrated that PRMT1 and PRMT5 had opposing effects on chemotherapeutic agent-mediated apoptosis in lung cancer cells. The identification of PRMT5 and PRMT1 as CFLAR_L_ regulators involved in cellular apoptosis may help in developing new strategies to increase the sensitivity of cancer cells to chemotherapy, which may eventually benefit lung cancer treatments.

## Additional file

Additional file 1: **Figure S1.** Interaction domains between CFLAR_L_ and PRMT5 and PRMT1**. A** HEK293FT cells were transfected with the pcDNA3.1-FLAG-CFLAR_L_ plasmids and co-transfected with all sections of PRMT5 and the control plasmid. Then, the cells were harvested and prepared for the IP assay after 16 h. The cells were treated with 20 μmol/L MG132 for 4 h. The precipitated proteins were analyzed by western blotting. **B** HEK293FT cells were transfected with the pcDNA3.1-FLAG-CFLAR_L_ plasmids and co-transfected with the section of PRMT1 and the control plasmid. Then, the cells were harvested and prepared for the IP assay after 16 h. The cells were treated with 20 μmol/L MG132 for 4 h. The precipitated proteins were analyzed by western blotting. **C** HEK293FT cells were transfected with the pcDNA3.1-MYC-PRMT5 plasmids and co-transfected with all sections of CFLAR_L_ and the control plasmid. The cells were the harvested and prepared for the IP assay after 20 h, and the precipitated proteins were analyzed by western blotting. **D** HEK293FT cells were transfected with the pcDNA3.1-MYC-PRMT1 plasmids and co-transfected with all sections of CFLAR_L_ and the control plasmid. The cells were harvested, prepared for the IP assay after 16 h, and treated with 20 μmol/L MG132 for 4 h. The expression of the corresponding protein was calculated as described in (C). **Figure S2.** PRMT5 and PRMT1 modulated apoptosis in NSCLC cells. **A** and **B** H460 cells were seeded in 6-well plates. PcDNA3.1-PRMT5 were transfected for 24 h. Cells were treated with pemetrexed [5.0 μM] for 48 h. Cells were collected for Flow Cytometry analysis. **C** and **D** H460 cells were seeded in 6-well plates. PRMT5 siRNA were transfected for 48 h. Cells were treated with pemetrexed [5.0 μM] for 48 h. Cells were collected for Flow Cytometry analysis. **E** and **F** A549 cells were seeded in 6-well plates. PRMT1 siRNA were transfected for 48 h. Cells were treated with pemetrexed [5.0 μM] for 48 h. Cells were collected for Flow Cytometry analysis. (DOCX 14 kb)

## Abbreviations

ADMA: Asymmetric dimethylation
CFLAR: CASP8 and FADD like apoptosis regulator
CHX: Chlorhexidine
DISC: death-inducing signaling complex
ITCH: Itchy E3 ubiquitin protain ligase
MMA: Monomethylation
NSCLC: non-small cell lung cancer
PARP: poly ADP-ribose polymerase
PRMT1: Protein arginine methyltransferase 1
PRMT5: Protein arginine methyltransferase 5
SDMA: Symmetric demethylation

## References

[1] Irmler M (1997). Inhibition of death receptor signals by cellular FLIP. Nature.

[2] Rasper DM (1998). Cell death attenuation by 'Usurpin', a mammalian DED-caspase homologue that precludes caspase-8 recruitment and activation by the CD-95 (Fas, APO-1) receptor complex. Cell Death Differ.

[3] Micheau O (2003). Cellular FLICE-inhibitory protein: an attractive therapeutic target?. Expert Opin Ther Targets.

[4] McLornan DP (2010). Prognostic significance of TRAIL signaling molecules in stage II and III colorectal cancer. Clin Cancer Res.

[5] Kreuz S, Siegmund D, Scheurich P, Wajant H (2001). NF-kappaB inducers upregulate cFLIP, a cycloheximide-sensitive inhibitor of death receptor signaling. Mol Cell Biol.

[6] Ricci MS (2004). Direct repression of FLIP expression by c-myc is a major determinant of TRAIL sensitivity. Mol Cell Biol.

[7] Skurk C (2004). The Akt-regulated forkhead transcription factor FOXO3a controls endothelial cell viability through modulation of the caspase-8 inhibitor FLIP. J Biol Chem.

[8] Zong H, Yin B, Chen J, Ma B, Cai D, He X (2009). Over-expression of c-FLIP confers the resistance to TRAIL-induced apoptosis on gallbladder carcinoma. Tohoku J Exp Med.

[9] Mawji IA (2007). A chemical screen identifies anisomycin as an anoikis sensitizer that functions by decreasing FLIP protein synthesis. Cancer Res.

[10] Poukkula M (2005). Rapid turnover of c-FLIPshort is determined by its unique C-terminal tail. J Biol Chem.

[11] Chang L (2006). The E3 ubiquitin ligase itch couples JNK activation to TNFalpha-induced cell death by inducing c-FLIP (L) turnover. Cell.

[12] Panner A, Crane CA, Weng C, Feletti A, Parsa AT, Pieper RO (2009). A novel PTEN-dependent link to ubiquitination controls FLIPS stability and TRAIL sensitivity in glioblastoma multiforme. Cancer Res.

[13] Panner A (2010). Ubiquitin-specific protease 8 links the PTEN-Akt-AIP4 pathway to the control of FLIPS stability and TRAIL sensitivity in glioblastoma multiforme. Cancer Res.

[14] Shi B, Tran T, Sobkoviak R, Pope RM (2009). Activation-induced degradation of FLIP (L) is mediated via the phosphatidylinositol 3-kinase/Akt signaling pathway in macrophages. J Biol Chem.

[15] Kerr E (2012). Identification of an acetylation-dependant Ku70/FLIP complex that regulates FLIP expression and HDAC inhibitor-induced apoptosis. Cell Death Differ.

[16] Getts RC, Stamato TD (1994). Absence of a Ku-like DNA end binding activity in the xrs double-strand DNA repair-deficient mutant. J Biol Chem.

[17] Tomimatsu N (2007). Ku70/80 modulates ATM and ATR signaling pathways in response to DNA double strand breaks. J Biol Chem.

[18] Fell VL, Schild-Poulter C (2015). The Ku heterodimer: function in DNA repair and beyond. Mutat Res Rev Mutat Res.

[19] Bedford MT (2007). Arginine methylation at a glance. J Cell Sci.

[20] Pal S, Sif S (2007). Interplay between chromatin remodelers and protein arginine methyltransferases. J Cell Physiol.

[21] Bedford MT, Richard S (2005). Arginine methylation an emerging regulator of protein function. Mol Cell.

[22] Gary JD, Clarke S (1998). RNA and protein interactions modulated by protein arginine methylation. Prog Nucleic Acid Res Mol Biol.

[23] Pal S, Vishwanath SN, Erdjument-Bromage H, Tempst P, Sif S (2004). Human SWI/SNF-associated PRMT5 methylates histone H3 arginine 8 and negatively regulates expression of ST7 and NM23 tumor suppressor genes. Mol Cell Biol.

[24] Wang L, Pal S, Sif S (2008). Protein arginine methyltransferase 5 suppresses the transcription of the RB family of tumor suppressors in leukemia and lymphoma cells. Mol Cell Biol.

[25] Pal S, Baiocchi RA, Byrd JC, Grever MR, Jacob ST, Sif S (2007). Low levels of miR-92b/96 induce PRMT5 translation and H3R8/H4R3 methylation in mantle cell lymphoma. EMBO J.

[26] Scoumanne A, Zhang J, Chen X (2009). PRMT5 is required for cell-cycle progression and p53 tumor suppressor function. Nucleic Acids Res.

[27] Jansson M (2008). Arginine methylation regulates the p53 response. Nat Cell Biol.

[28] Hsu JM (2011). Crosstalk between Arg 1175 methylation and Tyr 1173 phosphorylation negatively modulates EGFR-mediated ERK activation. Nat Cell Biol.

[29] Lin WJ, Gary JD, Yang MC, Clarke S, Herschman HR (1996). The mammalian immediate-early TIS21 protein and the leukemia-associated BTG1 protein interact with a protein-arginine N-methyltransferase. J Biol Chem.

[30] Yang Y, Bedford MT (2013). Protein arginine methyltransferases and cancer. Nat Rev Cancer.

[31] Lee J, Bedford MT (2002). PABP1 identified as an arginine methyltransferase substrate using high-density protein arrays. EMBO Rep.

[32] Zhang X, Cheng X (2003). Structure of the predominant protein arginine methyltransferase PRMT1 and analysis of its binding to substrate peptides. Structure.

[33] Cha B (2011). Methylation by protein arginine methyltransferase 1 increases stability of Axin, a negative regulator of Wnt signaling. Oncogene.

[34] Liu X, Yue P, Zhou Z, Khuri FR, Sun SY (2004). Death receptor regulation and celecoxib-induced apoptosis in human lung cancer cells. J Natl Cancer Inst.

[35] Liu X, Yue P, Khuri FR, Sun SY (2005). Decoy receptor 2 (DcR2) is a p53 target gene and regulates chemosensitivity. Cancer Res.

[36] Cui J, Wei M, Su X, Zhang Y, Su L, Liu X (2015). EHMT2 inhibitor BIX-01294 induces apoptosis through PMAIP1-USP9X-MCL1 axis in human bladder cancer cells. Cancer Cell Int.

[37] Hornbeck PV (2012). PhosphoSitePlus: a comprehensive resource for investigating the structure and function of experimentally determined post-translational modifications in man and mouse. Nucleic Acids Res.

[38] Jenuwein T, Allis CD (2001). Translating the histone code. Science.

[39] Strahl BD, Allis CD (2000). The language of covalent histone modifications. Nature.

[40] Lattouf H, Kassem L, Jacquemetton J, Choucair A, Poulard C, Trédan O (2019). LKB1 regulates PRMT5 activity in breast cancer. Int J Cancer.

[41] Cho JH, Lee MK, Yoon KW, Lee J, Cho SG, Choi EJ (2012). Arginine methylation-dependent regulation of ASK1 signaling by PRMT1. Cell Death Differ.

[42] Chang L, Kamata H, Solinas G, Luo JL, Maeda S, Venuprasad K, Liu YC, Karin M (2006). The E3 ubiquitin ligase itch couples JNK activation to TNFalpha-induced cell death by inducing c-FLIP (L) turnover. Cell.

[43] Hsu TS, Mo ST, Hsu PN, Lai MZ (2018). C-FLIP is a target of the E3 ligase deltex1 in gastric cancer. Cell Death Dis.

[44] McGowan EB, Becker E, Detwiler TC (1989). Inhibition of calpain in intact platelets by the thiol protease inhibitor E-64d. Biochem Biophys Res Commun.

[45] Ni H, Ren SY, Zhang LL, Sun Q, Tian T, Feng X (2013). Expression profiles of hippocampal regenerative sprouting-related genes and their regulation by E-64d in a developmental rat model of penicillin-induced recurrent epilepticus. Toxicol Lett.

[46] Yan F (2014). Genetic validation of the protein arginine methyltransferase PRMT5 as a candidate therapeutic target in glioblastoma. Cancer Res.

[47] Tsai WW, Niessen S, Goebel N, Yates JR, Guccione E, Montminy M (2013). PRMT5 modulates the metabolic response to fasting signals. Proc Natl Acad Sci U S A.

[48] Shilo K, et al. Cellular localization of protein arginine methyltransferase-5 correlates with grade of lung tumors. Diagn Pathol. 2013;8(201). 10.1186/1746-1596-8-201 PMC3933389 24326178

