# Supplementary material for: The arginine methyltransferase PRMT5 and PRMT1 distinctly regulate the degradation of anti-apoptotic protein CFLARL in human lung cancer cells
Source: J Exp Clin Cancer Res. 2019 Feb 8;38:64. doi: 10.1186/s13046-019-1064-8 (PMC6368745; doi:10.1186/s13046-019-1064-8)
Supplement: Supplementary file 1 — Figure S1. Interaction domains between CFLARL and PRMT5 and PRMT1. A HEK293FT cells were transfected with the pcDNA3.1-FLAG-CFLARL plasmids and co-transfected with all sections of PRMT5 and the control plasmid. Then, the cells were harvested and prepared for the IP assay after 16 h. The cells were treated with 20 μmol/L MG132 for 4 h. The precipitated proteins were analyzed by western blotting. B HEK293FT cells were transfected with the pcDNA3.1-FLAG-CFLARL plasmids and co-transfected with the section of PRMT1 and the control plasmid. Then, the cells were harvested and prepared for the IP assay after 16 h. The cells were treated with 20 μmol/L MG132 for 4 h. The precipitated proteins were analyzed by western blotting. C HEK293FT cells were transfected with the pcDNA3.1-MYC-PRMT5 plasmids and co-transfected with all sections of CFLARL and the control plasmid. The cells were the harvested and prepared for the IP assay after 20 h, and the precipitated proteins were analyzed by western blotting. D HEK293FT cells were transfected with the pcDNA3.1-MYC-PRMT1 plasmids and co-transfected with all sections of CFLARL and the control plasmid. The cells were harvested, prepared for the IP assay after 16 h, and treated with 20 μmol/L MG132 for 4 h. The expression of the corresponding protein was calculated as described in (C). Figure S2. PRMT5 and PRMT1 modulated apoptosis in NSCLC cells. A and B H460 cells were seeded in 6-well plates. PcDNA3.1-PRMT5 were transfected for 24 h. Cells were treated with pemetrexed [5.0 μM] for 48 h. Cells were collected for Flow Cytometry analysis. C and D H460 cells were seeded in 6-well plates. PRMT5 siRNA were transfected for 48 h. Cells were treated with pemetrexed [5.0 μM] for 48 h. Cells were collected for Flow Cytometry analysis. E and F A549 cells were seeded in 6-well plates. PRMT1 siRNA were transfected for 48 h. Cells were treated with pemetrexed [5.0 μM] for 48 h. Cells were collected for Flow Cytometry analysis. (DOCX 14 kb) [file 13046_2019_1064_MOESM1_ESM.docx]

**Supplementary Fig. S1. Interaction domains between CFLAR_L_ and PRMT5 and PRMT1. A** HEK293FT cells were transfected with the pcDNA3.1-FLAG-CFLAR_L_ plasmids and co-transfected with all sections of PRMT5 and the control plasmid. Then, the cells were harvested and prepared for the IP assay after 16 h. The cells were treated with 20 μmol/L MG132 for 4 h. The precipitated proteins were analyzed by western blotting. **B** HEK293FT cells were transfected with the pcDNA3.1-FLAG-CFLAR_L_ plasmids and co-transfected with the section of PRMT1 and the control plasmid. Then, the cells were harvested and prepared for the IP assay after 16 h. The cells were treated with 20 μmol/L MG132 for 4 h. The precipitated proteins were analyzed by western blotting. **C** HEK293FT cells were transfected with the pcDNA3.1-MYC-PRMT5 plasmids and co-transfected with all sections of CFLAR_L_ and the control plasmid. The cells were the harvested and prepared for the IP assay after 20 h, and the precipitated proteins were analyzed by western blotting. **D** HEK293FT cells were transfected with the pcDNA3.1-MYC-PRMT1 plasmids and co-transfected with all sections of CFLAR_L_ and the control plasmid. The cells were harvested, prepared for the IP assay after 16 h, and treated with 20 μmol/L MG132 for 4 h. The expression of the corresponding protein was calculated as described in (C).

**Supplementary Fig. S2. PRMT5 and PRMT1 modulated apoptosis in NSCLC cells. A** and **B** H460 cells were seeded in 6-well plates. PcDNA3.1-PRMT5 were transfected for 24h. Cells were treated with pemetrexed [5.0μM] for 48h. Cells were collected for Flow Cytometry analysis. **C** and **D** H460 cells were seeded in 6-well plates. PRMT5 siRNA were transfected for 48h. Cells were treated with pemetrexed [5.0μM] for 48h. Cells were collected for Flow Cytometry analysis. **E** and **F** A549 cells were seeded in 6-well plates. PRMT1 siRNA were transfected for 48h. Cells were treated with pemetrexed [5.0μM] for 48h. Cells were collected for Flow Cytometry analysis.
